# A dataset of sensory perception of chocolates, guacamoles, ice teas and crisps collected with consumers using six temporal methods

**DOI:** 10.1016/j.dib.2022.108708

**Published:** 2022-10-28

**Authors:** Michel Visalli, Sylvie Cordelle, Benjamin Mahieu, Catherine Pedron, Betty Hoffarth, Manon Praudel, Marine Coutière, Pascal Schlich

**Affiliations:** a Centre des Sciences du Goût et de l'Alimentation, AgroSup Dijon, CNRS, INRAE, Université Bourgogne Franche-Comté, F-21000 Dijon, France; b INRAE, PROBE research infrastructure, ChemoSens facility, F-21000 Dijon, France; c StatSC, ONIRIS, INRAE, Nantes, France

**Keywords:** TDS, TCATA, AEF, Free-comment, Sensometric

## Abstract

This article describes a dataset providing temporal sensory perception data of four dark chocolates, four guacamoles, four crisps and four ice teas collected from 436 consumers divided in six groups. Each group of consumers has tested all products using only one sensory evaluation method among: Temporal Dominance of Sensations (TDS, n=70), Temporal Check-All-That-Apply (TCATA, n=73), Attack-Evolution-Finish (AEF) dominance (n=74), AEF applicability (n=75), Free-Comment Attack-Evolution-Finish (FC-AEF) dominance (n=72) and FC-AEF applicability (n=72). Each consumer evaluated all the products: guacamoles and ice tea were evaluated in the lab in one session; chocolates and crisps were evaluated at home in two separate sessions. Within each product category, one sample has been replicated. The consumers started with product descriptions, then they gave a hedonic score, and after having tasted all the products related to a same category, they answered questions about product complexity and difficulty of the task. Consumer information included in the dataset is sex, age and frequency of consumption of each product category. This dataset is unique as it addresses several temporal methods applied on four product categories with different textures and levels of complexity. Thus, it could be very useful for the sensometric community to compare the different methods and their parameters: dominance vs. applicability, periods vs. continuous time, simultaneous vs. retrospective measures, list of terms vs. Free-Comment.

**Specifications Table**

**Table:** 

Subject	Food science
Specific subject area	Sensory analysis. Temporal methods.
Type of data	Tables Figures Questionnaire
How the data were acquired	Sensory data were acquired from six panels of consumers (436 consumers in total) at lab and at home using TimeSens© software 2.0 (INRAE, Dijon, France).
Data format	Raw Analyzed
Description of data collection	The consumers were randomly assigned (with the constraint of balance in gender and age between groups) to one of the six panels, each panel using a different method to describe the temporal perception of the products: • Temporal Dominance of Sensations – TDS, • Temporal Check All That apply – TCATA, • Attack-Evolution Finish dominance – AEF-D, • AEF applicability – AEF-A, • Free-Comment AEF dominance – FC-AEF-D, • FC-AEF-applicability – FC-AEF-A The four evaluated product categories (including various commercial brands) varied in composition, texture and sensory complexity. Crisps (solid) varied in fat and salt content; ice teas (liquid) varied in sugar/sweetener content, in flavor and variety of tea; guacamoles (semi-solid) varied in avocado and fat contents; chocolates (solid) varied in cocoa origin and content. Guacamoles and ice teas were evaluated at lab, crisps and chocolates at home. Hedonic data have been rated using 9-points discrete scales. The difficulty of the task has been scored on a 0-10 VAS scale. The items sensory complexity of each product category have been scored on 0-10 VAS scales.
Data source location	• City/Town/Region: Dijon and vicinity • Country: France
Data accessibility	The raw data, provided as a Microsoft Excel Worksheet, are available on the Mendeley data open-access research data repository. Repository name: Mendeley data Data identification number: 10.17632/fshtbhffth.1 Direct URL to data: *https://data.mendeley.com/datasets/fshtbhffth/1*
Related research article	

## Value of the Data

• These data are useful because they enable the comparison of six temporal methods applied on four product categories varying in sensory complexity, textures and compositions.
• The sensometric community can benefit from these data to compare different concepts: dominance vs. applicability, periods vs. continuous time, simultaneous vs. retrospective measures, list of terms vs. Free-Comment.
• These data can be reused to compare and document the sensory methods performances, to develop new statistical analyses or to study the links between perception, liking and complexity.

## 1 Objective

This dataset has been generated in order to compare the six sensory methods on different product spaces varying in complexity and sensory differences. It will serve as “material and methods” for several articles to come that will study temporal resolution, performances and drivers of liking. The ultimate objective is to conclude on the most appropriate method to use with consumers based on the type of product, location and expected level of performances (discrimination, repeatability, reproducibility).

## 2 Data Description

The dataset is provided as an Excel file (.xlsx) including 11 sheets.

The sheet “Consumer” provides information about the recruited consumers.

 “Panel” is the panel to which the consumer has been randomly assigned (TDS, TCATA, AEF_D, AEF_A, FC_AEF_D, FC_AEF_A).
 “Consumer” is the 3-character code of the consumer.
 “Gender” is the gender reported by the consumer (M for male or F for female).
 “Age” is the age range reported by the consumer (18_30: from 18 to 30 years old, 31_45: from 31 to 45 years old, 46_64: from 46 to 64 years old).
 “Consumption_IceTea”, “Consumption_Guacamole”, “Consumption_Chocolate”, “Consumption_Crisp” are the frequencies of consumptions of each product category (ice teas, guacamoles, dark chocolates, crisps) reported by the consumers (never, less than once a month, at least once a month, at least once a week).
 “Panel”, “Consumer” and “ProductCategory” columns are reported in each sheet following this one.
 The sheets “TDS” and “TCATA“ contain the temporal descriptions reported by the consumers of the panels of the same name.
 “Product” is the identifier of the product (character).
 “Time” is the time of each click on the attribute in seconds (numeric).
 “Attribute” is the code of the attribute (character).
 “Score” is 1 if “Attribute” has been considered dominant (TDS) or applicable (TCATA) by “Consumer” for “Product” during “Period” (numeric). For TCATA, an attribute remains applicable until the end or until deselected, in this case a new entry with score=0 is recorded.

The sheets “AEF_D” and “AEF_A“ contain the temporal descriptions reported by the consumers of the panels of the same name.

 “Product” is the identifier of the product (character).
 “Period” is the identifier of the period (A: attack, E: evolution, F: Finish).
 “Attribute” is the code of the attribute (character).
 “Score” is 1 if “Attribute” has been considered dominant (AEF_D) or applicable (AEF_A) by “Consumer” for “Product” during “Period”, 0 otherwise (numeric).

The sheets “FC_AEF_D” and “FC_AEF_A“ contain the temporal descriptions reported by the consumers of the panels of the same name.

 “Product” is the identifier of the product (character).
 “Period” is the identifier of the period (A: attack, E: evolution, F: Finish).
 “FrenchRawDescription” is the Free-Comment reported by the consumer (free text, in French).
 “EnglishRawDescription” is the English translation of “FrenchRawDescription” made using deepL translator (https://www.deepl.com/translator) and checked by the authors of this article.
 The sheet “Duration“ contains the durations of tasting of each “Product” by each “Consumer” from each “Panel”.
 “Duration” is the duration from the click on the start button to the click on the stop button, in seconds (numeric).

The sheet “Liking“ contains the liking scores reported for each “Product” by each “Consumer” from each “Panel”.

 “Liking” is the value rated on a discrete scale (numeric, between 1 and 9).

The sheet “Complexity“ contains the scores of the different items of the complexity questionnaire reported for each “ProductCategory” by each “Consumer” of each “Panel”. “Attribute” is the code of the item (IntensityOfDifferences, Familiarity, NumberOfSensations, EaseOfIdentification, Harmony, Balance, Persistence, Power, Complexity). “Score” is the score on the structured scale (numeric, between 0 and 10, precision of 0.01).

The sheet “Difficulty“ contains the scores of difficulty of the evaluation task reported for each context by each “Consumer” from each “Panel”. “Context” is the location of the measure (lab or home). “Score” is the score on the structured scale (numeric, between 0 and 10, precision of 0.01).

Table 1 summarizes the main characteristics of the six temporal methods.

Table 2 describes the product categories, codes and composition.

Table 3 reports the averaged scores of the items of the complexity questionnaire, by product category.

Table 4 summarizes the attributes used with the TDS, TCATA, AEF-A and AEF-D methods, by product category.

Table 5 reports the number of consumers having evaluated each product category, by panel.

Table 6 summarizes the individual characteristics of the consumers, by panel.

**Fig. 1:**
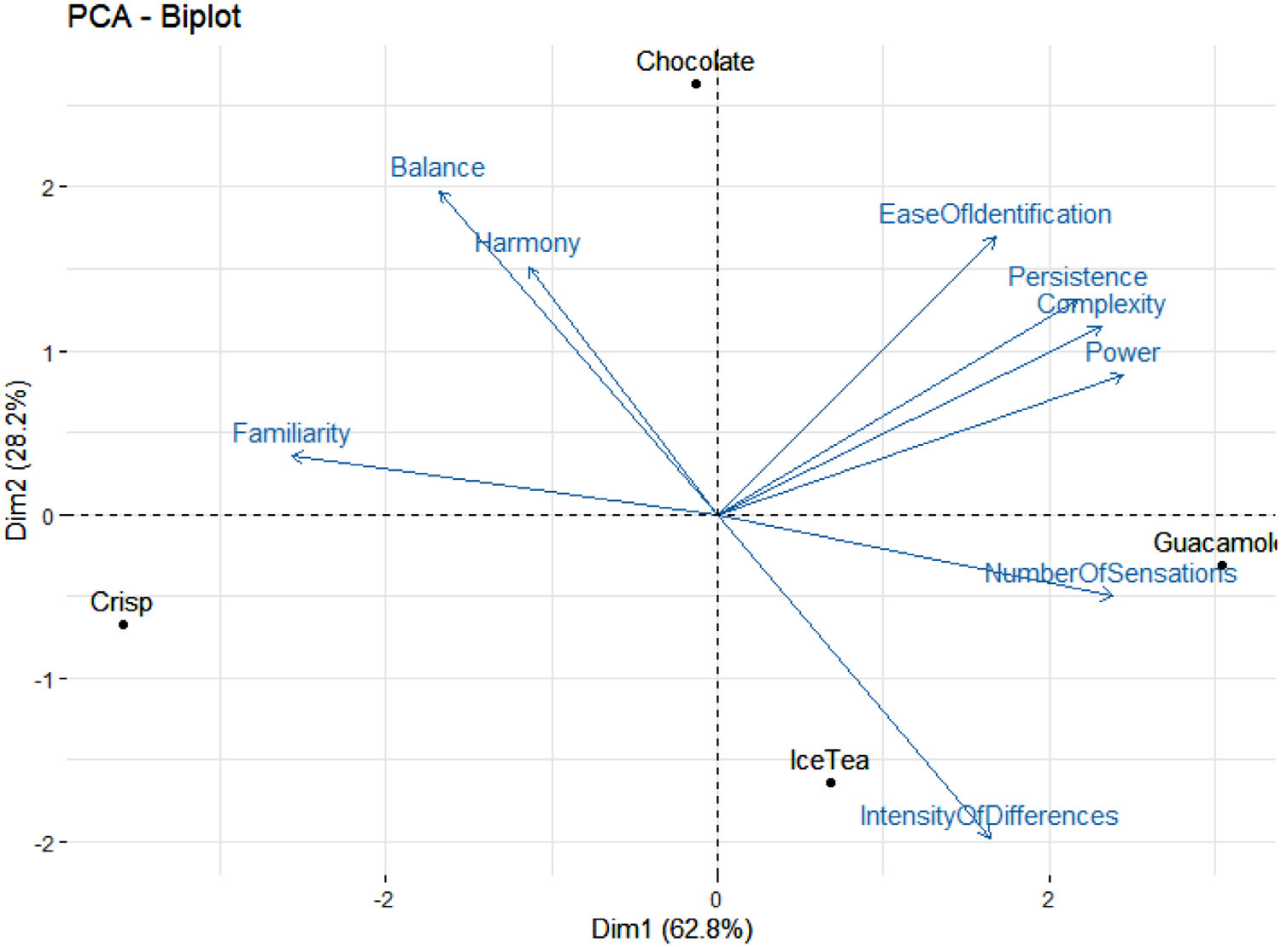
PCA of the averaged scores of the items of the complexity questionnaire by product category plotted using R 4.0.1 [7] and package FactoMineR [8].

Fig. 1 is the Principal Component Analysis of the averaged scores of the items of the complexity questionnaire by product category.

**Fig. 2:**
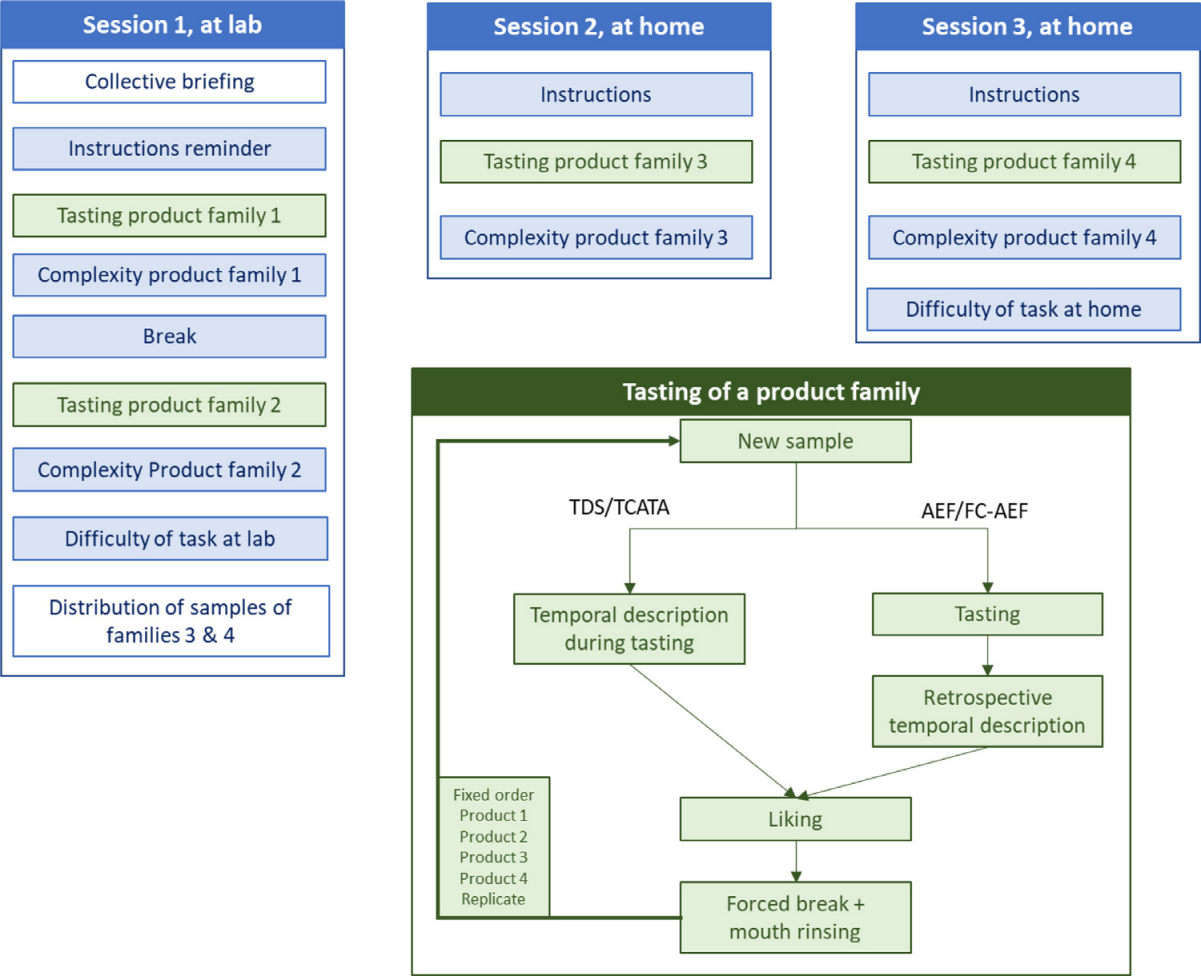
Experimental procedure chart. Steps in blue and green boxes were related to the TimeSens session. Steps in green boxes were repeated for each sample of each product category.

Fig. 2 is the experimental procedure chart.

Questionnaire includes commented screenshots of the online questionnaire (TimeSens V2 web app). It has been translated from French to English.

## 3 Experimental Design, Materials and Methods

### 3.1 Temporal methods

Six temporal methods were compared: Temporal Dominance of Sensations - TDS [1]; Temporal Check-All-That-Apply - TCATA [2]; Attack-Evolution-Finish - AEF [3] called here AEF-D (D for dominance); AEF-A (adaptation of AEF-D, the reported attributes being the applicable ones instead of the dominant ones); Free-Comment Attack-Evolution-Finish dominance - FC-AEF-D (an adaptation of FC-AEF [4], the reported attributes being the dominant ones instead of the applicable ones); FC-AEF, called here FC-AEF-A (A for Applicability).

**Table 1:** summary of the main characteristics of the six temporal methods.

Method	Moment of measure	Choice of attributes	Reported attributes	Temporal resolution
TDS	During tasting	Predefined list	Dominant ones, one at a time	Continuous
TCATA	During tasting	Predefined list	Applicable ones, zero, one or several at a time	Continuous
AEF-D	After tasting	Predefined list	Dominant ones, one at a time	Periods
AEF-A	After tasting	Predefined list	Applicable ones, zero, one or several at a time	Periods
FC-AEF-D	After tasting	Free-comment	Dominant ones, one at a time	Periods
FC-AEF-A	After tasting	Free-comment	Applicable ones, zero, one or several at a time	Periods

For AEF-D and FC-AEF-D, no definition was given for dominance, but the forced choice was supposed to imply the dominance concept.

### 3.2 Product categories and samples

In order to get more generalizable conclusions, the methods were compared on different product families varying in composition, sensory modalities and sensory complexity. The chosen products were commercial products accepted by a majority of consumers and easy to prepare for the experimenters. Bibliographical research on the products used in previous studies implementing these methods and pre-tests led to the selection of four product categories, each containing four variants. Crisps (solid, portion size: 3 g) varied in fat and salt, ice teas (liquid, portion size: 20 ml) varied in sugar/sweetener, flavor and variety of tea, guacamoles (semi-solid, portion size: 7 g) varied in avocado content and fat, and dark chocolates (solid, portion size: 10 g) varied in cocoa origin and content. To assess for individual and panel repeatability, one variant was replicated inside each product category.

**Table 2:** product categories, codes and composition (as reported on the packaging).

Product category	Codes	Composition
Crisp	C1, C1_rep (replicate of C1)	34 g fat, 1.3 g salt
Crisp	C2	23.9 g fat, 1.52 g salt
Crisp	C3	29 g fat, 1 g salt (sea salt)
Crisp	C4	34 g fat, 0.10 g salt
Guacamole	G1, G1_rep (replicate of G1)	92 % avocado, 16 g fat
Guacamole	G2	13 % avocado, 9.5 g fat
Guacamole	G3	90 % avocado, 14.6 g fat
Guacamole	G4	95 % avocado, 18 g fat
IceTea	IT1	4.7 g sugar, black tea, white peach
IceTea	IT2	4.5 g sugar, white tea, peach and rosemary
IceTea	IT3, IT3_rep (replicate of IT3)	4.3 g sugar, sweeteners, black tea, peach
IceTea	IT4	0 g sugar, sweeteners, black tea, peach
Chocolate	CH1	85 % cocoa, origin Madagascar
Chocolate	CH2	80 % cocoa, origin Equator
Chocolate	CH3	70 % cocoa, origin Peru
Chocolate	CH4, CH4_rep (replicate of CH4)	74 % cocoa, origin Côte d'Ivoire

The ice teas and guacamoles were stored in the refrigerator at 4°C until tasting. They were taken out of the refrigerator a few minutes before the test so as not to be too fresh when tasted.

The samples were evaluated according to a between-subjects design (the treatment being the temporal method), with products evaluated in a fixed order, which is quite unconventional in sensory analysis. Indeed, this study was purely methodological, and one of the objectives was to compare temporal methods (not products) regarding to individual differences. In this specific case, it is common to fix the order of testing to minimize the variance introduced by different orders of presentation across the subjects [5,6]. By doing so, it must be remembered before drawing any product dependent conclusions that an order effect may have affected the product comparison. Since there was no indication of which order would minimize this potential product order effect, the presentation rank of each product was determined randomly, with the exception of the second evaluation of the repeated sample which was always presented at the fifth rank. The position of the product in the presentation design was indicated in the code (example: G1 was served at position 1). The samples were labelled using random 3-digits codes.

**Table 3:** averaged scores of the items of the complexity questionnaire by product category.

	Balance	Complexity	Ease of identification	Familiarity	Harmony	Intensity of differences	Number of sensations	Persistence	Power
Chocolate	5.58	5.86	5.48	6.15	5.77	5.81	5.23	6.70	6.82
Crisp	5.38	4.47	4.79	6.66	5.82	6.38	4.87	5.58	5.60
Guacamole	4.92	6.01	5.23	5.37	5.69	7.56	6.77	6.99	7.18
IceTea	4.96	5.37	5.19	5.84	5.39	7.36	5.62	6.00	6.51

Table 3 summarizes the results of the complexity questionnaire.

Fig. 1 shows the correlations between the different dimensions of the complexity questionnaire.

### 3.3 Attributes

A pre-test carried out by an internal panel of six persons familiar with temporal methods was carried out to select the descriptors for the TDS, TCATA, AEF-D and AEF-A methods.

**Table 4:** attributes used with the TDS, TCATA, AEF-D and AEF-A methods, by product category.

Product category	Sensory modality	French word	English translation
IceTea, Guacamole, Chocolate	Basic taste	Acide	Sour
IceTea, Guacamole, Chocolate	Basic taste	Sucré	Sweet
IceTea, Guacamole, Chocolate	Basic taste	Amer	Bitter
IceTea	Mouthfeel	Astringent/Râpeux	Astringent/Rough
IceTea	Flavor	Pêche	Peach
IceTea, Chocolate	Flavor	Chimique	Artificial
IceTea	Flavor	Thé	Tea
IceTea	Texture	Aqueux/Dilué	Watery/Diluted
Guacamole	Texture	Onctueux/Gras	Smooth/Fat
Guacamole	Texture	Fluide	Fluid
Guacamole	Texture	Épais	Thick
Guacamole	Flavor	Citronné	Lemon
Guacamole	Flavor	Avocat	Avocado
Guacamole	Flavor	Pimenté	Spicy
Guacamole	Flavor	Oignon/Échalote	Onion/Shallot
Guacamole	Flavor	Tomate	Tomato
Guacamole	Flavor	Herbes aromatiques	Aromatic/Herb
Crisp	Texture	Craquant/Dur	Crackly/Hard
Crisp	Texture	Croustillant	Crispy
Crisp	Texture	Collant/Pâteux	Sticky/Pasty
Crisp	Texture	Fondant	Melting
Crisp	Basic taste	Salé	Salty
Crisp	Flavor/Mouthfeel	Gras	Fat
Crisp	Flavor	Pomme de terre	Potato
Crisp	Flavor	Grillé	Roasted
Crisp	Flavor	Fade	Bland
Chocolate	Texture	Sec/Poudreux	Dry/Powdery
Chocolate	Texture	Fondant/Gras	Melting/Fat
Chocolate	Texture	Collant	Sticky
Chocolate	Mouthfeel	Astringent	Astringent
Chocolate	Flavor	Cacao	Cocoa
Chocolate	Flavor	Fruité	Fruity
Chocolate	Flavor	Floral	Floral
Chocolate	Flavor	Boisé/Torréfié	Woody/Roasted

The descriptors were presented in a random order on the screen but this order was constant for each consumer across the evaluations of a same product category. Due to a technical problem, only nine attributes over twelve have been presented to the AEF-A panel on the Chocolate product category.

### 3.4 Consumers

At the moment of the recruitment, the objective was to have a minimum size of 64 consumers for each panel, or 384 subjects in total. Taking into consideration the possible withdrawals, 504 consumers were preselected from a population registered in the ChemoSens Platform's PanelSens database. This database has been declared to the relevant authority (Commission Nationale Informatique et Libertés—CNIL—n° d'autorisation 1148039). The selection criteria included gender, age and frequency of consumption of the product categories. Restrictions related to the health context related to COVID-19 were added to the usual restrictions. People with an allergy, people on a restrictive diet, people considered vulnerable and pregnant women were therefore excluded. A total of 436 people participated in this study. They were compensated for their participation with vouchers worth 15 euros.

The consumers were randomly assigned to one of the six panels with a constraint of balance in gender and age between panels. Each panel used a different temporal method to describe the temporal evolution of the samples from each product category over three sessions, one at lab and two at home, on different days.

**Table 5:** number of consumers having evaluated each product category, by panel.

Context	Product category	Panel TDS	Panel TCATA	Panel AEF-D	Panel AEF-A	Panel FC-AEF-D	Panel FC-AEF-A
Lab, 1 session	IceTea	70	73	74	75	72	72
	Guacamole						
Home, 2 days	Crisp	70	72	74	75	66	70
	Chocolate	68	72	71	75	68	70

**Table 6:** individual characteristics of the consumers, by panel. Cells indicate percentages.

Individual characteristics	Panel TDS	Panel TCATA	Panel AEF-D	Panel AEF-A	Panel FC-AEF-D	Panel FC-AEF-A
Age: 18-30	20.00	21.92	21.62	14.67	20.83	18.06
Age: 31-45	35.71	31.51	36.49	42.67	41.67	37.50
Age: 46-64	44.29	46.58	41.89	42.67	37.50	44.44
Gender: male	68.57	72.60	70.27	70.67	66.67	68.06
Gender: female	31.43	27.40	29.73	29.33	33.33	31.94
IceTea: at least once a month	42.86	26.03	37.84	41.33	45.83	38.89
IceTea: at least once a week	25.71	36.99	28.38	21.33	18.06	22.22
IceTea: less than once a month	27.14	36.99	31.08	36.00	30.56	34.72
IceTea: never	4.29	0.00	2.70	1.33	5.56	4.17
Guacamole: at least once a month	57.14	47.95	43.24	41.33	47.22	38.89
Guacamole: at least once a week	14.29	10.96	9.46	13.33	9.72	12.50
Guacamole: less than once a month	28.57	36.99	43.24	44.00	41.67	47.22
Guacamole: never	0.00	4.11	4.05	1.33	1.39	1.39
Chocolate: at least once a month	21.43	12.33	24.32	16.00	13.89	12.50
Chocolate: at least once a week	71.43	79.45	59.46	70.67	70.83	75.00
Chocolate: less than once a month	2.86	6.85	12.16	10.67	11.11	4.17
Chocolate: never	1.43	0.00	0.00	2.67	1.39	2.78
Crisp: at least once a month	32.86	38.36	40.54	54.67	40.28	44.44
Crisp: at least once a week	48.57	45.21	36.49	32.00	40.28	26.39
Crisp: less than once a month	14.29	15.07	22.97	12.00	16.67	20.83
Crisp: never	0.00	0.00	0.00	1.33	0.00	0.00

## 4 Experimental Procedure

### 4.1 Collective briefing (session 1)

To start, all consumers were invited to a collective briefing at lab in groups of 16. The temporal method was explained and the attributes were presented. A demonstration of the task was done by the panel leader by video projecting the screens (see attached questionnaire, TDS: screens 1-27, TCATA: screens 28-54, AEF-D/A: screens 55-83, FC-AEF-D/A: screens 84-112) of the software (TimeSens V2, [9]). Then, the consumers were encouraged to ask questions about the task before taking place in individual booths.

### 4.2 Instructions for temporal description

The instructions, depending on the temporal method, were reminded on the screen. The screen was displayed during a minimum time of 30 seconds to ensure the consumers read the instructions. The instructions were quite similar for all product categories, below are instructions for IceTea.

TDS instructions (screen 3): “For each ice tea, you will proceed as follows. You will take a sip, while simultaneously pressing the “Mouthing” button. A list of buttons will be displayed on the screen. Throughout the tasting of the sip, as soon as you perceive a dominant sensation, you will have to press the button corresponding to this sensation. Some sensations may never be selected, others may be selected multiple times during the tasting of the sip. You will continue to indicate the sensations perceived after swallowing the sip. When you no longer perceive anything, you will click on “I don't perceive anything anymore” button. You should only taste one sip of each sample of ice tea. Familiarize yourself with the sensations available and their location on the screen before putting the product in your mouth.”

TCATA instructions (screen 30): “For each ice tea, you will proceed as follows. You will take a sip, while simultaneously pressing the “Mouthing” button. A list of checkboxes will be displayed on the screen. Throughout the tasting of the sip, as soon as you perceive a sensation, you will have to check the checkbox corresponding to this sensation. You will need to uncheck the checkbox as soon as you no longer perceive this sensation. Some sensations may never be selected, others may be selected multiple times during the tasting of the sip. You will continue to check the attributes perceived and uncheck the attributes no longer perceived after swallowing the sip. When you no longer perceive anything, you will click on the “I don't perceive anything anymore” button. You should taste only one sip of each sample of ice tea. Familiarize yourself with the sensations available and their location on the screen before putting the product in your mouth.”

AEF (D and A) instructions (screen 57): “For each ice tea, you will proceed as follows. You will take a sip, while simultaneously pressing the “Mouthing” button. When you no longer perceive anything, you will press the “Next” button. At this moment, we will ask you to describe your perception by choosing, from a list of terms, a sensation [AEF-D] / one or several sensations [AEF-A] for each period of your perception: beginning, middle and end. Some sensations may never be selected, others may be selected in several periods. An example is given to you on the following page. You should taste only one sip of each sample of ice tea.

FC-AEF (D and A) instructions (screen 86): “For each ice tea, you will proceed as follows. You will take a sip, while simultaneously pressing the “Mouthing” button. When you no longer perceive anything, you will press the “Next” button. At this moment, we will ask you to describe, using your own words, the sensation [FC-AEF-D] / sensation(s) [FC-AEF-A] you experienced for each period of your perception: beginning, middle and end. An example is given to you on the following page. Use only words, don't make sentences. Compound words and expressions are allowed. Example: "long in the mouth". You should taste only one sip of each sample of ice tea.”

### 4.3 Tasting, descriptive and hedonic evaluation of the five ice tea samples

The consumers had to evaluate the products of category IceTea under red light. They first had to check the sample code (TDS: screen 4, TCATA: screen 31, AEF-D/A: screen 59, FC-AEF-D/A: screen 88). Consumers of AEF (screen 60) and FC-AEF (screen 89) panels started with a screen with no attribute, allowing to record the duration of the tasting. The measurement screens were displayed just after, depending on the temporal method (TDS: screen 5, TCATA: screen 32, AEF-D/A: screen 61, FC-AEF-D/A: screen 90). After having described their perception, whatever the panel, the consumers had to rate their preference for the tasted sample on a 9-point discrete scale (TDS: screen 6, TCATA: screen 33, AEF-D/A: screen 62, FC-AEF-D/A: screen 91). After that, a 30-second forced break (TDS: screen 7, TCATA: screen 34, AEF-D/A: screen 63, FC-AEF-D/A: screen 92) was imposed, inviting the consumers to rinse their mouth with mineral water. The procedure was repeated for the three other samples and the replicated one.

### 4.4 Evaluation of the complexity of the ice tea product category

After the evaluation of the five samples, the consumers had to evaluate the complexity of the product category, using an adaptation of the questionnaire of [10]. Nine items were evaluated over four consecutive screens (TDS: screens 9-12, TCATA: screens 36-39, AEF-D/A: screens 65-68, FC-AEF-D/A: screens 94-97) using structured scales. Items included, in this order: “Intensity of differences”, “Familiarity”, “Number of perceived sensations” “Easiness of identification of sensations”, “Harmony between sensations”, “Balance”, “Persistence”, “Power” and “Overall complexity”.

### 4.5 Evaluation of the five guacamole samples

After a 5-minute forced break (TDS: screen 13, TCATA: screen 40, AEF-D/A: screen 69, FC-AEF-D/A: screen 98), the five guacamole samples were evaluated following the procedure described in sections 5.3 and 5.4.

### 4.6 Participants characteristics and frequency of consumption

Participants characteristics and frequency of consumption were asked (TDS: screen 16, TCATA: screen 43, AEF-D/A: screen 72, FC-AEF-D/A: screen 101) in order to check consistency with answers collected during the recruitment phase.

### 4.7 Evaluation of the difficulty of the task at lab

To end the lab session, the consumers had to evaluate the difficulty of the temporal description task using a structured scale (TDS: screen 17, TCATA: screen 44, AEF-D/A: screen 73, FC-AEF-D/A: screen 102).

### 4.8 End of lab session and preparation of home session

The duration of the session was approximately 45 minutes. After that, each consumer had to take home samples from the two other product categories: Chocolates, and Crisps. The samples of crisps were stored in individual disposable plastic cups with lids. The samples of chocolate were packed into aluminum foils. All the samples were put into a bag and the consumers were asked to keep the bag at room temperature.

The consumers were informed that they would receive two separated e-mails inviting them to connect to the internet session using a browser (TDS: screen 18, TCATA: screen 45, AEF-D/A: screen 74, FC-AEF-D/A: screen 103). There was one mail for each product category, and the sessions were still designed using TimeSens V2. The consumers were instructed to evaluate all the samples of a product category on a same day, but the two categories on different days.

### 4.9 Evaluation of the five samples of crisps, at home (session 2)

The consumers received the first mail the same day of the tasting in lab. They were instructed to do the session 2 in the same day, or if unable to do so, the next morning. In session 2, they had to evaluate the samples of crisps following the procedure described in sections 5.3, 5.4, and 5.6.

### 4.10 Evaluation of the five samples of chocolate, at home (session 3)

A second email was sent to the consumers the day after they completed session 2. In session 3, they had to evaluate the samples of chocolate following the procedure described in sections 5.3, 5.4, and 5.6. Then, a final question was asked about the usefulness of having done the task at lab before doing the test at home (TDS: screen 27, TCATA: screen 54, AEF-D/A: screen 83, FC-AEF-D/A: screen 112). They had to answer on a structured scale.

The whole procedure was summarized in Fig. 2 below.

## Ethics Statements

Each participant was informed of the conditions for participating and had to validate a consent form.

## CRediT Author Statement

**Sylvie Cordelle:** Methodology, Validation, Investigation, Resources, Project administration, Writing – review & editing; **Marine Coutière:** Methodology, Investigation, Resources, Data curation; **Betty Hoffarth:** Resources; **Benjamin Mahieu:** Methodology, Writing – review & editing; **Catherine Pedron:** Resources; **Manon Praudel:** Methodology, Resources, Data Curation; **Pascal Schlich:** Conceptualization, Methodology, Supervision, Funding acquisition, Writing – review & editing; **Michel Visalli:** Conceptualization, Methodology, Software, Validation, Formal analysis, Data curation, Writing – original draft, Visualization.

## Declaration of Competing Interest

The authors declare that they have no known competing financial interests or personal relationships that could have appeared to influence the work reported in this paper.

The authors declare the following financial interests/personal relationships which may be considered as potential competing interests:

## Data Availability

A dataset of sensory perception of chocolates, guacamoles, ice teas and crisps collected with consumers using six temporal methods (Original data) (Mendeley Data).
